# Ubiquitin-proteasomal degradation of antiapoptotic survivin facilitates induction of apoptosis in prostate cancer cells by pristimerin

**DOI:** 10.3892/ijo.2014.2561

**Published:** 2014-07-25

**Authors:** YONG BO LIU, XIAOHUA GAO, DORAH DEEB, CHRIS BRIGOLIN, YIGUAN ZHANG, JIAJIU SHAW, KIRIT PINDOLIA, SUBHASH C. GAUTAM

**Affiliations:** 1 Department of Surgery, Henry Ford Health System, Detroit, MI 48202, USA; 2 Department of Medical Genetics, Henry Ford Health System, Detroit, MI 48202, USA; 3 Department of Internal Medicine, Henry Ford Health System, Detroit, MI 48202, USA

**Keywords:** pristimerin, prostate cancer, apoptosis, survivin, ubiquitin, proteasome

## Abstract

Pristimerin (PM), a quinonemethide triterpenoid, is a promising anticancer agent with potent antiproliferative and apoptosis-inducing activities against cancer cell lines. However, the anticancer activity and mechanisms of PM in prostate cancer cells have not been adequately investigated. Here we report that the degradation of survivin plays an important role in the antiproliferative and proapoptotic effects of PM in carcinoma of the prostate (CaP) cell lines. Treatment with PM inhibited proliferation and induced apoptosis in LNCaP and PC-3 cells as characterized by the loss of cell viability and an increase in Annexin V-binding and cleavage of PARP-1, respectively. The antiproliferative and apoptosis-inducing effects of PM were associated with the inhibition of cell cycle regulatory proteins, antiapoptotic survivin and members of the Bcl-2 family. Data showed that response to PM is regulated by survivin since overexpression of survivin rendered CaP cells resistant to PM. Furthermore, downregulation of survivin by PM was mediated through the ubiquitin-proteasomal degradation. Together, these data demonstrate that pristimerin inhibits proliferation and induces apoptosis in CaP cells by abolishing survivin through the ubiquitin-proteasome pathway.

## Introduction

Herbal remedies are commonly used in traditional medicine to treat and prevent human diseases including cancer. Numerous plant derived flavonoids and phenolic/polyphenolic compounds with antioxidant and anti-inflammatory activities are currently used by cancer patients as dietary supplements to complement chemotherapy. In fact, isolation and identification of bioactive components from medicinal plants have led to the synthesis of potent anticancer drugs, including Vinca alkaloids, taxol, camptothecan, etoposide and retinoids. Triterpenoids are members of a large family of structurally related compounds known as cyclosqualenoids that are widely distributed in nature. Pristimerin (PM) is a quinonemethide triterpenoid present in various plant species in the Celastraceae and Hippocrateaceae families (1,2). PM and related compounds have shown anti-inflammatory, antioxidant and antimalarial activities (3–5). PM has also shown potent antiproliferative and apoptosis-inducing activity in glioma, leukemia, breast, lung and prostate cancer cell lines (6–9). Induction of apoptosis by PM involves generation of reactive oxygen species (ROS), activation of caspases, mitochondrial dysfunction, inhibition of nuclear factor κB (NF-κB), Akt and MAP kinases (10–13). In addition, PM also inhibits proteasome activity, tumor cell migration and angiogenesis (8,14,15).

Carcinoma of the prostate is the most commonly diagnosed cancer in American males and the second ranked cause of cancer related mortality (16). An estimated 233,000 new cases of prostate cancer will be diagnosed and 29,480 deaths are expected to occur from this disease in the United States in 2014 (17). Current therapies (e.g., androgen deprivation, radical prostatectomy, radiotherapy or brachytherapy) while effective in treating localized prostate cancer have limited efficacy against advanced disease and metastatic hormone-refractory disease remains incurable (18–20). Since the incidence of CaP increases with advancing age, prostate cancer is expected to be an increasingly greater problem as life expectancy improves.

In a previous report we have shown that PM induces apoptosis in CaP cell lines through a ROS-dependent Bcl-2 degradation pathway (21). In the present study, we demonstrate that induction of apoptosis in CaP cells by PM is associated with inhibition of cell cycle regulatory proteins and proteasomal degradation of antiapoptotic survivin, a member of the inhibitors of apoptosis (IAP) family.

## Materials and methods

### Reagents

PM was purchased from Sigma Chemicals (St. Louis, MO). Anti-PARP-1, anti-Bcl-2, anti-Bcl-xL and anti-survivin antibodies were purchased from Santa Cruz Biotechnology, Inc. (Santa Cruz, CA). The 96 AQueous One Solution Proliferation Assay System was from Promega (Madison, WI). Annexin V-FITC apoptosis detection kit was purchased from BD Pharmingen (San Diego, CA). Stock solution of PM (100 mM) was prepared in DMSO and all test concentrations were prepared by diluting stock solution in tissue culture medium.

### Cell lines

LNCaP and PC-3 human prostate cancer cell lines were obtained from the American Type Culture Collection (ATCC, Rockville, MD). LNCaP were grown in RPMI-1640 supplemented with FBS and penicillin/streptomycin. PC-3 cells were grown in F-12K nutrient mixture (Gibco BRL, Rockville, MD) supplemented with 10% fetal calf serum, 1% penicillin/streptomycin, and 25 mM HEPES buffer. Both cell lines were cultured at 37°C in a humidified atmosphere consisting of 5% CO_2_ and 95% air, and maintained by subculturing cells twice a week.

### Measurement of cell viability (MTS assay)

Tumor cells (1×10^4^) in 100 μl of tissue culture medium were seeded into each well of a 96-well plate. After 24-h incubation to allow cells to adhere, cells were treated with PM at concentrations ranging from 0 to 5 μM. Cultures were incubated for additional 72 h and cell viability was then determined by the colorimetric MTS assay using CellTiter 96 AQueous One Solution Proliferation Assay System from Promega. This assay measures the bioreduction of the tetrazolium compound MTS by intracellular dehydrogenases in the presence of electron-coupling reagent phenazine methosulfate. MTS and phenazine methosulfate were added to the culture wells, and cultures were incubated for 2 h at 37°C. The absorbance, which is directly proportional to the number of viable cells in the cultures, was measured at 490 nm using a microplate reader.

### Apoptosis assay

Apoptosis was assessed by the binding of Annexin V-FITC to phosphotidylserine, which is externalized to the outer leaflet of the plasma membrane early during induction of apoptosis. Briefly, untreated cells and cells treated with PM were resuspended in the binding buffer provided in the Annexin V-FITC apoptosis detection kit II (BD Biosciences, San Diego, CA) and allowed to react with 5 μl of Annexin V-FITC reagent and 5 μl of propidium iodide for 30 min at room temperature in the dark. Stained cells were analyzed by flow cytometry using Accuri C6 flow cytometer (Accuri Cytometers Inc., Ann Arbor, MI). The induction of apoptosis by PM was confirmed by the cleavage of PARP-1 by western blot analysis.

### Western blot analysis

Cell lysates were prepared by detergent lysis [1% Triton-X 100 (v/v), 10 mM Tris-HCl (pH 7.5), 5 mM EDTA, 150 mM NaCl, 10% glycerol, 2 mM sodium vanadate, 5 μg/ml leupeptin, 1 μg/ml aprotinin, 1 μg/ml pepstatin A and 10 μg/ml 4-2-aminoethyl-benzenesulfinyl fluoride]. Lysates were clarified by centrifugation at 14,000 × g for 10 min at 4°C, and protein concentrations were determined by Bradford assay. Samples (50 μg) were boiled in an equal volume of sample buffer [20% glycerol, 4% SDS, 0.2% bromophenol blue, 125 mM Tris-HCl (pH 7.5), and 640 mM 2-mercaptoethanol] and separated on 10% SDS-polyacrilamide gels. Proteins resolved on the gels were transferred to nitrocellulose membranes and probed with antibodies against proteins of interest followed by HRP-conjugated secondary antibody. Immune complexes were visualized by chemiluminescence. Protein bands were imaged and band densities analyzed using the NIH/Scion image analysis software.

### DNA transfection

For expression of HA tagged-survivin, semi-confluent cultures of PC-3 cells in 60 mm^2^ cell culture dishes were transfected with 10 μg of empty or HA-survivin expression vector (pcDNA3-HA-survivin) (CH3 BioSystems, Amherst, NY) using Lipofectamine Plus reagent. After incubation for 36 h, overexpression of survivin in transfected cells was confirmed by immunoblotting.

### Immunoprecipitation

After treatment with PM (5 μM) for 6 h cells were washed with cold PBS and lysed in NP-40 cell lysis buffer (Invitrogen, Camarillo, CA) supplemented with 2 mM sodium vanadate, 5 μg/ml leupeptin, 1 μg/ml aprotinin, 1 μg/ml pepstatinin, and 10 μg/ml 4-2-aminoethyl-benzenesulfinyl fluoride for 30 min on ice. Supernatants were collected after centrifugation at 14,000 × g for 10 min and protein concentration was determined. Each sample (400 μg protein) in 200 μl of antibody binding buffer containing anti-HA antibody was incubated for 1 h at room temperature followed by incubation with protein A agarose beads for 1 h. Immune complexes were washed two times with lysis buffer and analyzed for ubiquitin by western blot analysis.

### Statistical analysis

Data are expressed as mean ± SD. The difference between control and treatment groups was determined using Dunnett’s multiple comparison test. Differences with p<0.05 were considered statistically significant.

## Results

### Pristimerin inhibits proliferation of CaP cells

**Figure 1:**
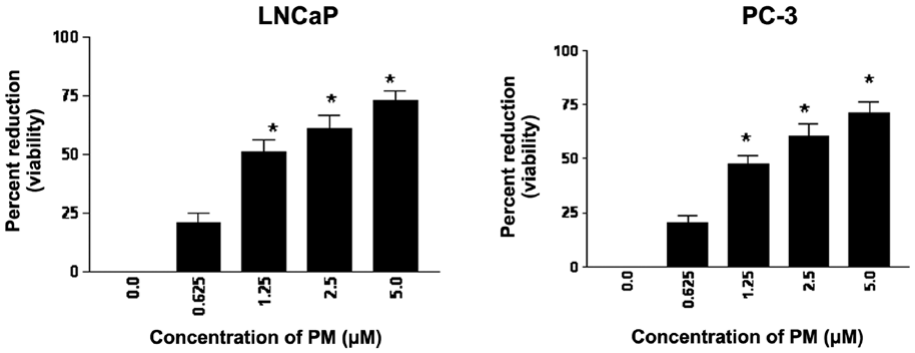
Pristimerin inhibits proliferation of CaP cells. LNCaP and PC-3 cells (1×10^4^/well) were seeded in a 96-well plate for 24 h. Cells were treated with PM at concentrations ranging from 0 to 5 μM for 72 h in triplicate. Cell viability was measured by MTS assay using CellTiter AQueous Assay System. ^*^p<0.05 compared to control cells. Experiment was repeated three times.

The effect of PM on proliferation of CaP cells (LNCaP and PC-3 cells) was examined using MTS assay. For this, cells were treated with PM at concentrations of 0 to 5 μM for 72 h and the viability of cultures was determined. As shown in Fig. 1, measurable reduction in viability (~20%) was observed in both cell lines at 0.625 μM PM; however, significant reduction in viability occurred at 1.25 to 5 μM PM (47–73%, p<0.05). Thus, PM reduced the proliferation both androgen-sensitive (LNCaP) and androgen-resistant (PC-3) CaP cells.

### PM inhibits cell cycle regulatory proteins in CaP cells

**Figure 2:**
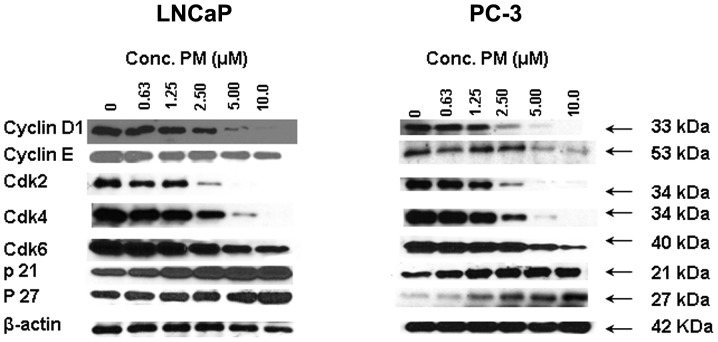
Effect of pristimerin on cell cycle regulatory proteins. LNCaP and PC-3 cells were treated with PM as above and cell lysates were analyzed for levels of cyclin D1, cyclin E, cdk2, cdk4, cdk6, p21 and p27 by western blot analysis. Each experiment was repeated three times.

Since cell division is regulated by cyclins and cyclin-dependent kinases (cdks) and cdk inhibitors such as WAF1/21 and KIP1/27, we investigated the effect of PM on these cell cycle regulators. For this, LNCaP and PC-3 cells were treated with PM (0–10 μM) for 24 h and levels of cyclin D1, cyclin E, cdk2, cdk4, cdk6, p21 and p27 were analyzed by western blot analysis. As shown in Fig. 2, treatment with PM reduced the level of cyclin D1 and cyclin E in LNCaP cells, whereas only cyclin E was reduced in PC-3 cells. On the other hand, levels of cdk2, cdk4 and cdk6 were reduced in both cell lines in a dose-related manner. Contrary to the inhibition of cyclin D1 cyclin E and cdks 2, 4 and 6 treatment with PM increased the levels of cdk inhibitors p21 and p27. Thus, inhibition of cyclin D1 and E and cdks 2, 4 and 6 suggests arrest of LNCaP and PC-3 cells in G0/G1 cell cycle phase.

### PM induces apoptosis in CaP cells

**Figure 3:**
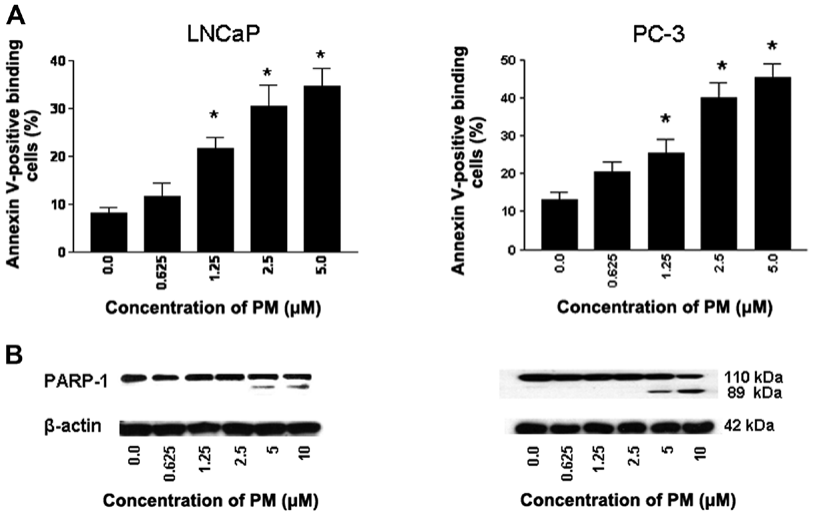
Pristimerin induces apoptosis. (A) Annexin V-FITC binding. LNCaP and PC-3 cells were treated with PM at 0 to 5 μM for 24 h and then reacted with 5 μl of Annexin V-FITC and 5 μl propidium iodide for 30 min and the percentage of Annexin V-FITC binding cells was determined by flow cytometry. (B) Cleavage of PARP-1. Tumor cells were treated with PM as above and PARP-1 was analyzed by immunoblot analysis. Similar results were obtained in 3 independent experiments. ^*^p<05 compared to control cells (no PM).

Whether inhibition of proliferation of CaP cells by PM leads to induction of apoptosis was investigated next. Thus, LNCaP and PC-3 cells were treated with PM (0 to 5 μM) for 24 h and induction of apoptosis was measured from the binding of Annexin V-FITC by flow cytometry and cleavage of PARP-1 by western blot analysis. As shown in Fig. 3A, only a small percentage of untreated LNCaP and PC-3 cells bound Annexin V-FITC (8 to 12%, respectively). In contrast, the percentage of Annexin V-FITC binding cells (both cell lines) increased in a dose-dependent manner after treatment with PM at 0.625 to 5 μM (LNCaP, 11–32%; PC-3, 19–43%).

The induction of apoptosis was confirmed by the cleavage of PARP-1 as identified by decrease in 110 kDa native protein and the emergence of an 89 kDa cleaved PARP-1 fragment in both cell lines treated with PM (Fig. 3B). Thus, increase in Annexin V-FITC-binding and the cleavage of PARP-1 demonstrated induction of apoptosis by PM.

### PM inhibits apoptosis-related proteins in CaP cells

**Figure 4:**
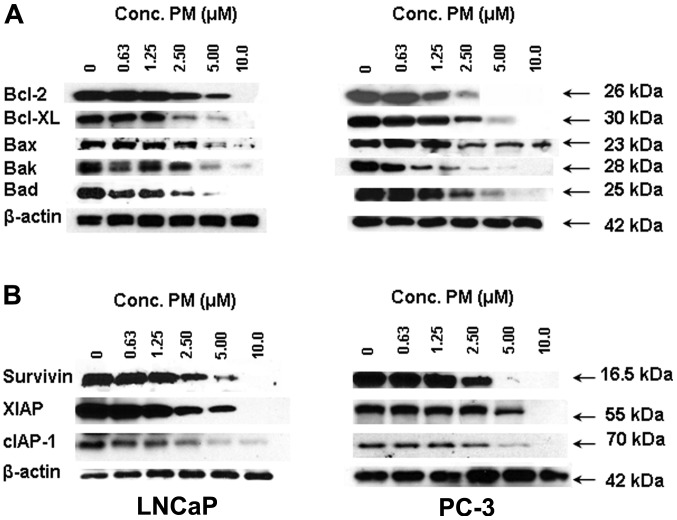
Effect of pristimerin on Bcl-2 and IAP family proteins. LNCaP and PC-3 cells were treated with PM at 0 to 10 μM for 24 h and cell lysates were analyzed for (A) Bcl-2 family members Bcl-2, Bcl-xL, Bax, Bak, Bad and (B) IAP family members survivin, XIAP and cIAP-1 by western blot analysis. Each measurement was repeated two times.

Apotosis is regulated by a number of pro and anti-apoptotic cellular proteins belonging to the Bcl-2 and IAP families of proteins. To ascertain the effect of PM on these apoptosis-regulatory proteins we analyzed the levels of some of the more prominent members of the Bcl-2 and IAP families by western blot analysis. Treatment with PM (0–10 μM) decreased the levels of antiapoptic Bcl-2, Bcl-xL and proapoptotic Bax, Bak and Bad in a dose-dependent manner in both cell lines (Fig. 4A). A similar inhibitory effect of PM on the expression of antiapoptotic IAP family members, such as survivin, XIAP and cIAP-1 was observed (Fig. 4B).

### Survivin regulates response to PM in CaP cells

**Figure 5:**
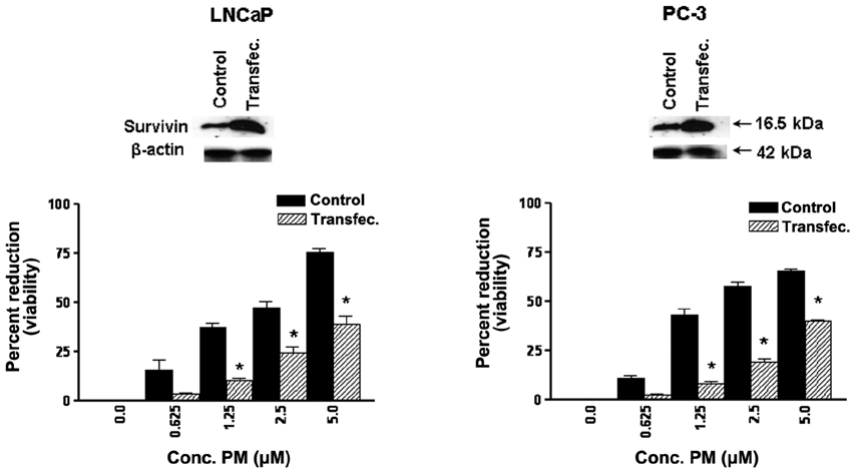
Overexpression of survivin reduces response to pristimerin. LNCaP and PC-3 cells were transfected with survivn expression plasmid as described in Materials and methods and overexpression of survivin was confirmed by western blot analysis (insets). Response to PM (0–5 μM) was measured in 72 h MTS assay as described in Fig. 1. ^*^p<0.05 compared to control cells. Experiment was repeated three times.

Survivin is an IAP family member that plays an important role in cell cycle regulation and inhibition of apoptosis. To examine the relevance of survivin in antitumor activity of PM, we measured the response of CaP cells overexpressing surviving to PM in MTS assay. For this, LNCaP and CaP cells were transfected with survivin expression vector (pcDNA3-HA-survivin) and after confirming overabundance of survivin in transfected cells their response to PM was measured in 72 h MTS assay. As shown in Fig. 5, there was significant reduction in the sensitivity of transfected cells to PM compared to control cells. Transfection with empty plasmid showed no change in response to PM (not shown). These data indicated that survivin plays an important role in the response of CaP cells to PM.

### Pristimerin downregulates survivin through ubiquitin-proteasome degradation pathway

**Figure 6:**
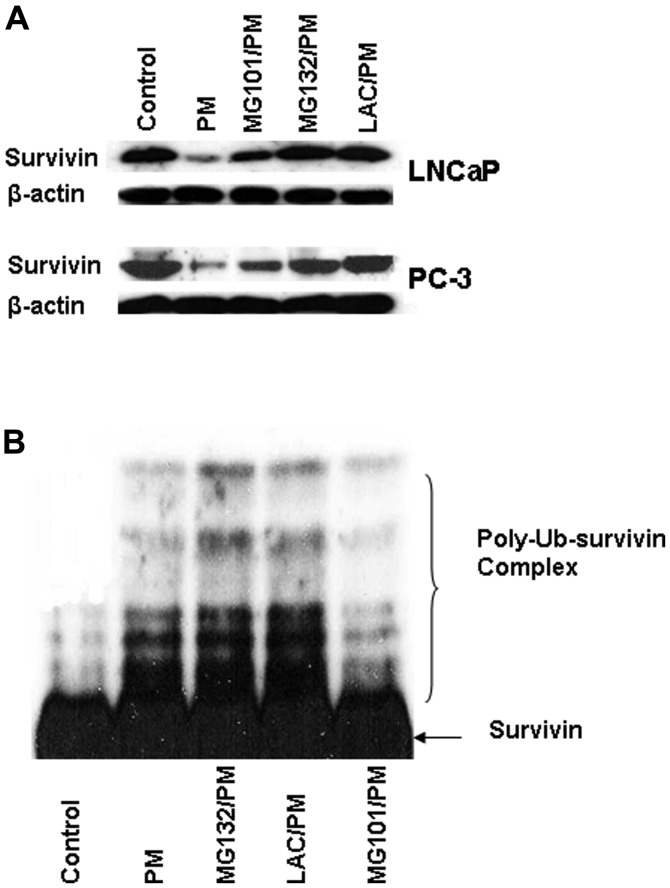
Pristimerin downregulates survivin through ubiquitin-proteasomal degradation pathway. (A) Effect of proteasome inhibitors. LNCaP and PC-3 cells were treated with PM (5 μM) in the presence or absence of proteasomal inhibitors MG132 (20 μM) and lactacystin (10 μM) or calpain inhibitor MG101 (20 μM) for 24 h and expression of survivin was analyzed by western blot analysis. (B) Effect on ubiquitination of survivin. PC-3 cells expressing HA-survivin were treated with PM (5 μM) for 6 h in the presence or absence of proteasome inhibitors MG132 (20 μM) and LAC (10 μM) or calpain inhibitor MG101 (20 μM) for 6 h. Cell lysates were immunoprecipitated with anti-HA antibody and immune complexes were analyzed for ubiquitin by western blot analysis. Similar results were obtained in two independent experiments.

To obtain insight into the mechanism by which PM reduces survivin in CaP cells we investigated the role of ubiquitin-proteasome degradation pathway in downregulation of survivin. First, the effect of calpain inhibitor MG101 and proteasome inhibitors MG132 and lactacystin on PM-mediated downregulation of survivin was examined. As shown in Fig. 6A, treatment with lysosomal protease inhibitor MG101 only partially reversed the inhibition of survivin by PM (~30% reversal). In contrast, pretreatment with proteasome inhibitors MG132 and lactacystin completely blocked the inhibition of survivin by PM (Fig. 6A).

The result above indicated the involvement of proteasome in PM-induced downregulation of survivin. To further establish the role of proteasome in degradation of survivin, we investigated the effect of PM on ubiquitination of survivin. For this, PC-3 cells transfected with survivin expression vector (pcDNA3-HA-survivin) were treated with PM for 6 h in the presence or absence of proteasome inhibitors MG132, lactacystin or caplain inhibitor MG101. Cells were treated with PM for 6 h because treatment for 6 h was found to induce maximal ubiquitination of survivin. Cell lysates were subjected to immunoprecipitation with anti-HA antibody followed by immunoblotting with anti-ubiquitin antibody to detect ubiquitinated survivin products. As shown in Fig. 6B, treatment with PM alone induced ubiquitination of survivin; however, treatment with PM in the presence of proteasome inhibitors MG132 and LAC resulted in additional accumulation of the polyubiquitinated survivin products. On the other hand, treatment with PM in the presence of calpain inhibitor MG101 did not cause accumulation of the polyubiquitinated survivin products. Taken together, these data indicated that survivin downregulation by PM is mediated through an ubiquitin-proteasomal degradation pathway.

## Discussion

There is intense interest in developing novel agents and treatment strategies to treat hormone-refractory metastatic prostate cancer. We and others have shown that PM exhibits potent antitumor activity against a wide range of cancer cell lines, including prostate cancer cells through multiple mechanisms (6–14,20). However, the significance of survivin, a potent inhibitor of apoptosis and a regulator of cell division in mediating response to PM in cancer cells has not been investigated. Thus, the present study was undertaken to examine the role of survivin in apoptotic cell death of prostate cancer cells by PM. Our results demonstrated the antiproliferative activity of PM both in androgen-sensitive and androgen-refractory prostate cancer cells. This result suggested that the inhibition of tumor cell proliferation by PM may be attributable to cell cycle inhibition. Indeed, PM has been shown to arrest cell cycle in G0/G1 phase in pancreatic cancer cells (21). Cell cycle progression is controlled by cyclins, cyclin-dependent kinases (cdks) and cdk inhibitors. In the present study, although a formal cell cycle analysis was not performed, treatment with PM downregulated levels of cyclin D1 and E in both CaP cell lines. PM also inhibited the expression of cdk2, cdk4 and cdk6 in both cell lines. Cyclin D1 and E in conjunction with cdk2, cdk4 and cdk6 regulate cell cycle progression through G1 phase. Thus, inhibition of cyclin D1 and E and cdk2, cdk4 and cdk6 suggests that PM might inhibit proliferation by arresting prostate cancer cells in G1-phase. Data also suggest that increase in expression of the cdk inhibitors p21 and p27 by PM may also facilitate G1 arrest by inhibiting the activity of cyclinE-cdk2 complexes that promotes G1-S phase progression.

In most instances, inhibition of cell proliferation by anticancer agents forces tumor cells to undergo apoptosis. PM increased Annexin V binding and cleaved PARP-1 in both cell lines indicating induction of apoptosis. This result corroborates our previous findings demonstrating induction of apoptosis by PM in epithelially-derived ovarian and pancreatic cancer cells via the inhibition of antiapoptotic (prosurvival) signaling molecules such as Akt, NF-κB and mTOR (14,20). In these tumor systems, induction of apoptosis involved cleavage of caspases-8, -9 and -3, loss of mitochondrial membrane potential and generation of free radicals supporting results of studies reported by others.

The intrinsic (mitochondrial) pathway of apoptosis is regulated by members of the Bcl-2 family of proteins that includes both pro- and anti-apoptotic molecules (22). In addition, members of the IAP family are potent inhibitors of apoptosis (23). Bcl-2 and Bcl-xL are two major antiapoptotic Bcl-2 family members that reside in the mitochondrial membrane and inhibit apoptosis by preventing the activation of inner mitochondrial permeability transition pore and release of proapotogenic mitochondrial contents including cytochrome *c* (24). PM inhibited Bcl-2 and Bcl-xL in both cell lines in a dose-related manner (Fig. 4). Interestingly, proapoptotic Bax, Bak and Bad were also inhibited by PM. Normally, proapoptotic Bax, Bak and Bad counteract antiapoptotic Bcl-2 and Bcl-xL and if the ratio of the antiapoptotic and proapoptotic members is tilted in favor of proapoptotic proteins, apoptosis ensues. Since PM reduced both anti- and pro-apoptotic Bcl-2 family members the exact role of Bcl-2 family of proteins in induction of apoptosis by PM in prostate cancer cells remains unresolved.

cIAP-1, XIAP and survivin are members of the inhibitor of apoptosis family of proteins (IAP) that block apoptosis by blocking activation or neutralizing the activity of caspases 3, 7 and 9 (23,25). cIAP-1 interferes with the activation of caspases, whereas XIAP binds to and inhibits caspase 3, 7 and 9. Survivin also inhibits caspase activation. Treatment with PM reduced the expression of these IAP members in prostate cancer cells, thereby contributing to the induction of apoptosis by PM.

Besides inhibiting apoptosis, survivin also regulates cell division and cytokinesis (26,27). Survivin is only expressed in the G2-M phase and during mitosis it localizes to the mitotic spindle by interaction with tubulin. Because of the prominent role survivin plays in the inhibition of apoptosis and regulation of cell division, we investigated the significance of survivin in mediating response to PM and the mechanism by which PM down-regulates its expression in CaP cells. The former was addressed by evaluating the response of tumor cells expressing abundance of survivin. Overexpression of survivin increased the resistance of tumor cells to PM (Fig. 5), implicating survivin in mediating the response to PM.

Levels of many short-lived proteins associated with apoptosis and cell cycle including survivin are regulated by ubiquitin-proteosome degradation pathway (28,29). Whether PM-mediated reduction in levels of survivin occurred through protesomal degradation was examined using pharmacological inhibitors of proteasomes. As shown in Fig. 6, proteasome inhibitors MG132 and lactacystin completely blocked the inhibition of survivin by PM whereas calpain inhibitor MG101 only partially reversed the inhibitory effect of PM, indicating that degradation of survivin by PM is primarily by proteasomes.

The degradation of proteins by 26S proteasome requires ubiquitination of target proteins through addition of multiple ubiquitin moieties at lysine residues. To confirm the involvement of proteasomes in PM-induced degradation of surviving, we analyzed ubiquitin-survivin complexes in tumor cells treated PM in the presence of proteasome inhibitors MG132 and lactacystin or calpain inhibitor MG101. Treatment with PM in the presence of MG132 or LAC resulted in accumulation of polyubiquitinated survivin products compared to treatment with PM alone. On the other hand, treatment with PM in the presence of MG101 did not cause accumulation of polyubiquitinated survivin. Taken together, these data demonstrated that downregulation of survivin by PM is mediated through the ubiquitin-proteasome degradation pathway. Thus, understanding the role and mechanism by which PM downregulates survivin may facilitate development of PM for the prevention/treatment of prostate cancer.
